# Supplementary material for: Are carbon emissions trading and green financial instruments synergistic? -Comprehensive quantitative research based on content analysis
Source: PLoS One. 2024 Mar 7;19(3):e0298601. doi: 10.1371/journal.pone.0298601 (PMC10919601; doi:10.1371/journal.pone.0298601)
Supplement: S1 Data — (ZIP) [file pone.0298601.s001.zip › Data/地级市Z_F工作报告.zip!/╔╜╢1⁄2/╟α╡║╩╨/└┤╫╘╣1⁄2╓┌║┼í╛┬φ┐╦╩2╛▌═°í┐_2012.docx]

各位代表：

　　现在，我代表市人民政府向大会报告工作，请予审议，并请各位政协委员和其他列席人员提出意见。

　　一、第十四届政府工作回顾

　　2008年市第十四届人民政府组成以来，在中共青岛市委的坚强领导下，深入贯彻科学发展观，大力实施“环湾保护、拥湾发展”战略，积极作为，科学务实，有效应对国际金融危机的冲击，改革开放和现代化建设取得重大成就。2011年是“十二五”的开局之年，全市上下解放思想，凝心聚力，抢抓蓝色经济区建设重大战略机遇，坚持以世界眼光谋划未来，以国际标准提升工作，以本土优势彰显特色，深入开展寻标、对标、达标、夺标活动，在重大战略实施、重点区域开发、重点领域改革、重点项目推进等方面取得新的突破，再次荣获全国文明城市称号，在建设现代化国际城市的进程中迈出坚实步伐。

　　四年来，全市生产总值跨越三个千亿元台阶，2011年达到6615.6亿元，年均增长12.5%；地方财政一般预算收入达到566亿元，年均增长17.9%；全社会固定资产投资达到3502.5亿元，年均增长23.5%；社会消费品零售总额达到2233亿元，年均增长16.4%；城市居民人均可支配收入达到28567元，年均增长12.5%；农民人均纯收入达到12370元，年均增长13.3%。提前完成了“十一五”节能减排目标。

　　（一）加快转变发展方式，经济实力显著增强

　　现代服务业快速发展。服务业增加值占生产总值比重达到47.8%，提高4.8个百分点，成为国家首批服务业综合改革试点城市。引进金融机构40家，金融业增加值占服务业增加值比重达到10.1%。港口吞吐量和集装箱吞吐量分别达到3.8亿吨、1300万标箱，空港旅客吞吐量超过1170万人次。

　　接待国内外游客超过5000万人次，旅游业总收入超过680亿元。规模以上工业总产值达到1.27万亿元，形成了六个先进制造业千亿级产业集群。启动老城区企业搬迁72户，23户竣工投产。战略性新兴产业产值突破2000亿元，占规模以上工业比重达到17.3%。粮食总产达到363万吨，建成粮食、蔬菜两个百万亩示范区，现代农业稳步发展。自主创新能力进一步增强，获得国家科技成果奖43项。新增中国驰名商标39个，取得高速动车、液晶模组等重大创新成果，国家认定企业技术中心、国家工程技术研究中心分别达到21家、7家，发明专利申请量和授权量分别增长4.2倍、3.5倍，成为国家创新型试点城市和首个技术创新工程试点城市。引进各类人才14.4万人。积极发展循环经济，新天地静脉产业园列入国家首批“城市矿产”示范基地。中德生态园开工建设。市区污水集中处理率达到88%，提高23个百分点。全市森林覆盖率达到37.8%，建成区绿化覆盖率达到43.8%，分别提高4个和2.3个百分点，成为全国绿化模范城市。

　　（二）推动区域协调发展，城乡面貌明显改善

　　城市功能不断增强。胶州湾隧道、胶州湾大桥、环湾大道等项目竣工通车，城市地铁、铁路青岛北客站开工建设。蓝色经济区建设加快推进，西海岸经济新区和蓝色硅谷发展规划编制完成，高新区、董家口港区建设初具规模，海洋科学与技术国家实验室一期、国家生物产业基地医药中试中心建成，国家深海基地、海洋科考船、山东大学青岛校区等重点项目顺利推进。实施数字化、网格化管理模式，提升了城市管理水平。市容环境十大整治行动成效显著。世园会筹办工作全面加速。县域工业化和城镇化步伐加快，城镇化率达到67.8%。各级财政投入“三农”资金282.4亿元，是上一个五年的1.9倍。农村“五化工程”基本完成，大沽河综合治理工程开工建设，农村饮水安全、库区移民后期扶持等工作取得新的成效，农村生产生活条件得到明显改善。

　　（三）切实保障和改善民生，社会保持和谐稳定

　　民生质量持续改善。市级财政对社会民生投入401.5亿元，占预算支出的54%，年均增长26.4%。城乡义务教育和特殊教育实行免费，完成550所薄弱中小学改造，高等教育毛入学率达到46%。新农合筹资标准从每人每年100元提高到260元，基本药物制度覆盖政府办基层医疗卫生机构，基本和重大公共卫生服务覆盖城乡居民。县级公立医院改革试点启动。国医堂、妇女儿童医院、北部中心医院、西海岸医院等建成使用，市第三人民医院迁建、妇女儿童医院二期等工程开工建设。完成了第六次人口普查，人口自然增长率控制在5‰以内。城镇登记失业率控制在3%以内。社会保障体系实现全覆盖，企业退休人员养老金提高59%，城乡低保标准分别提高40%和73.6%。建立了多层次住房保障体系，解决了15万户家庭的住房困难。强化了稳物价、控房价的政策措施。建立了社会救助和保障标准与物价上涨联动机制。市区新增集中供热面积4124万平方米，普及率达到70%。市区管道燃气气化率达到78%。大力发展公共交通，环保公交车超过87%，市政设施进一步完善。城乡和谐社区建设深入推进，社会治安环境持续优化。实施安全生产网格化监管，遏制了重特大事故发生。健全了食品安全管理体制机制，市区全部批发市场、70%的零售市场建成肉类流通追溯体系。健全突发事件处置机制，城市应急能力明显提高。

　　（四）实施文化强市战略，文化事业健康发展

　　公共文化服务网络实现城乡全覆盖，建成青岛大剧院等一批文化设施，全市44处公共图书馆、文化馆、博物馆免费开放，获得首批创建国家公共文化服务体系示范区资格。建成30个文化创意产业园区，华强文化科技产业园一期等项目开业，文化创意产业增加值占生产总值的7.6%。获得国家版权示范城市称号。开展了第三轮全国文明城市创建工作，文明城市复查测评总成绩居省会和副省级城市首位。军民携手取得抗击浒苔自然灾害的重大胜利，成功举办了奥帆赛、残奥帆赛，圆满完成海军节、十一运等重大任务。国防建设得到加强，军政军民关系更加密切，连续第七次荣获“全国双拥模范城”称号。提前一年完成对口支援北川灾后重建任务。民族、宗教、外事、侨务、对台、气象、地震、人防、统计、档案、史志和仲裁等工作得到加强，妇女、儿童、青少年、老龄、红十字、慈善、残疾人等事业全面发展。

　　（五）深入推进改革开放，发展活力不断增强

　　重点领域和关键环节改革稳步推进。整合组建4家政府投资公司，规范了市区两级融资平台。国家级开发区体制机制创新和小城镇改革试点取得积极进展。大力扶持中小企业，促进民营经济健康发展，民营经济增加值占生产总值比重达到53.5%。新增9家上市企业，融资109亿元。组建广电影视、报业、出版、演艺、网络五大国有文化传媒集团，文化体制改革实现突破。对外开放进一步扩大。实现进出口总额2236亿美元，实际利用外资110亿美元，引进内资2320亿元，新增世界500强企业16家，境外投资14.8亿美元，服务外包执行额连年倍增。我市成为港澳与内地紧密经贸关系合作示范城市、国家电子商务示范城市。前湾保税港区封关运营，口岸大通关明显提速。国际友好城市达到57个。

　　（六）努力加强政府自身建设，公共服务能力不断提升

　　政府服务效能进一步提高。自觉接受市人大及其常委会的法律监督和工作监督，主动接受市政协的民主监督，广泛听取民主党派、工商联和无党派人士的意见。向市人大常委会提交地方性法规草案19件，制定政府规章16件，办理人大代表议案建议2625件，政协提案建议3232件。完成新一轮政府机构改革。深化行政审批制度改革，建成市行政审批服务大厅和公共资源交易大厅，行政许可事项和非行政许可审批事项分别削减70%、66%，审批效率平均提速65%。推行相对集中行政处罚权和综合执法试点，实现网上规范透明运行。完善城市规划委员会和专家咨询委员会制度。政府门户网站保持全国前列，政府信息公开制度基本形成。加强惩治和预防腐败体系建设，扩大行政监察和审计监督覆盖面。“小金库”和商业贿赂治理取得明显成效。连续三年开展向市民报告、听市民意见、请市民评议活动，构建了公众评价政府绩效的新机制。

　　各位代表！过去四年的成绩来之不易。这是党中央国务院、省委省政府和中共青岛市委正确领导的结果，是市人大、市政协和社会各界有效监督、大力支持的结果，是全市人民团结拼搏、共同奋斗的结果。在此，我代表市第十四届人民政府，向全市人民，向人大代表和政协委员，向中央、省驻青单位，向驻青部队和武警官兵，向社会各界人士和离退休老同志，向所有关心支持青岛建设发展的海内外朋友们，表示衷心的感谢和崇高的敬意!

　　总结过去，着眼未来，按照科学发展观的要求，以世界眼光、国际标准来衡量，我们还面临不少困难和挑战，许多工作需要改进和加强。主要表现在：经济规模还不够大，城市竞争力还不强，创新创业环境有待改善，转方式调结构的步伐需要加快；城镇化水平还不高，城市布局结构有待优化，全域统筹发展的措施需要加强；城乡居民收入水平还不高，社会事业发展相对滞后，保障和改善民生、公共服务均等化还需要更大力度；政府职能转变仍不到位，政府工作人员视野不宽、标准不高、能力不强的问题不同程度地存在，极少数政府工作人员甚至以权谋私、贪污腐败，造成恶劣影响，政府自身建设任重而道远。这些问题都必须认真加以解决。

　　二、今后五年的目标任务

　　各位代表！市第十一次党代会绘就了青岛未来发展的宏伟蓝图，开启了率先科学发展、实现蓝色跨越，加快建设宜居幸福现代化国际城市的新征程，为政府工作指明了方向。今后五年，既要看到外部环境复杂多变、面临诸多困难和挑战，也要看到青岛独特的区位条件、良好的发展基础和难得的历史机遇，我们将承前启后、责无旁贷地担负起时代赋予的庄严使命，谱写青岛发展的新篇章。

　　今后五年，要高举中国特色社会主义伟大旗帜，以邓小平理论和“三个代表”重要思想为指导，深入贯彻落实科学发展观，紧紧围绕科学发展这个主题和加快转变经济发展方式这条主线，抢抓山东半岛蓝色经济区建设的重大机遇，全面贯彻落实市第十一次党代会精神，以世界眼光谋划未来，以国际标准提升工作，以本土优势彰显特色，率先科学发展、实现蓝色跨越，加快建设宜居幸福的现代化国际城市，为建设经济文化强省作出更大贡献。

　　今后五年，经济社会发展的主要预期目标是：全市生产总值年均增长11%，地方财政一般预算收入年均增长15%，城市居民人均可支配收入和农民人均纯收入年均增长11%，城镇化率达到76%。主要目标的确定，与“十二五”规划目标相衔接，又体现市第十一次党代会提出的基本要求，在实际工作中，要努力发展得更好更快，力争在经济规模上再造一个新青岛。

　　（一）加快转变发展方式，建立更具特色的产业体系

　　突出蓝色，强化蓝色经济引领作用。加快西海岸经济新区开发建设，再创港口、园区、产业发展优势，推动青岛港向世界第一大港迈进，构筑创新开放的现代化国际新城区，成为蓝色跨越的引擎。加快推进蓝色硅谷建设，打造国际一流的海洋科技研发中心、成果孵化中心、人才集聚中心和海洋新兴产业培育中心。深化蓝色经济区改革发展试点，构建特色鲜明、国内领先的现代海洋产业体系，海洋经济增加值争取达到1500亿元，占生产总值比重达到12.5%，建设蓝色青岛，加快向全国蓝色经济领军城市迈进。

　　突出高端，加快产业转型步伐。以高端技术、高端项目和高新企业为载体，大力发展高端制造业、高端服务业和高端农业，促进实体经济提质增效。发展高端制造，改造传统工业，培育十条千亿级先进制造业产业链。推进服务经济跨越发展，加快建设东北亚重要的区域性服务业中心城市。坚持高端农业引领，提升农业产业化、专业化、标准化、品牌化、国际化水平，率先基本实现农业现代化。

　　突出新兴，培育新的支柱产业。加快开发信息、生物、材料、能源、高端装备、节能环保新技术，加强规划引领和政策支持，突破核心技术，培育龙头项目，促进云计算、新能源汽车等新兴产业规模化发展。推进三网融合，加快光纤入户，建设智慧城市。规划建设一批新兴产业功能区，形成具有创新能力、竞争力强的企业集群。战略性新兴产业增加值占生产总值比重达到16%，加快形成支柱能力。

　　突出创新，建设创新型城市。大力发展创新型经济，构建完善的科技创新体系，形成企业主导、市场运作、产学研资深度合作的创新模式。加大投入，加快公共服务平台和孵化器建设。实施人才强市战略，营造聚才创业的良好环境，造就一批高水平的创新型科技领军人才、技能大师和创新团队，促进城市向创新创业、内生活力增长型转变。

　　（二）加快优化空间布局，构建更具活力的发展格局

　　实施全域统筹、三城联动、轴带展开、生态间隔、组团发展战略，发展海湾型都市，建设大青岛。全域统筹，以四方、李沧、平度、莱西和全域镇村建设为重点，推进七区统筹和城乡统筹，加快北部区域崛起，缩小南北差距、城乡差距。三城联动，做优做美东岸城区，做大做强西岸城区，做高做新北岸城区，打造功能互补、相互依托、各具特色的都市新区。轴带展开，以大沽河为生态中轴，建设贯穿南北的防洪屏障、生态长廊、现代农业聚集带、特色城镇与新型农村社区示范区；沿滨海蓝色经济发展带、烟威青综合发展带和济潍青综合发展带梯次推进，纵深拓展。组团发展，重点加快董家口港城、鳌山湾新城、新河新城、姜山新城、空港新城、北客站枢纽、世园生态都市新区以及红岛区域组团建设。加快市域轨道交通等骨干路网建设，支撑组团互动发展。

　　突出特色，优化产业空间布局，打造区域经济板块。市区重点建设高端服务业聚集区，着力打造现代服务业高地。规划建设金融中心、总部商务、现代物流、旅游会展、文化创意、软件外包、科技孵化等十项千万平方米高端服务业工程。按照国际标准，市南区要发展好金融商务、总部经济、时尚商业和商务旅游；市北区要建设好邮轮母港、啤酒文化和高端商贸聚集区；四方区要抓好科技研发、文化创意、滨海旅游和商务发展区建设；李沧区要建设好交通商务区、生态都市新区和商贸聚集区；崂山区要建设好金融新区、会展旅游和信息服务园区；黄岛区要建设好保税物流和国际经济合作示范区，发展度假旅游、信息服务和文化创意；城阳区要集约发展企业总部、商贸物流、广告创意；高新区重在规模化发展软件外包、研发孵化、高端制造，建设高新技术密集区和科技、生态、人文新城。五市以先进制造业为主攻方向，担负起全市工业发展的重任。开发建设胶南董家口临港重工业发展区、胶州高端制造聚集区、即墨蓝色制造聚集区、平度新河生态科技产业新城和莱西姜山新城，扩展五市省级开发区承载能力，向高新技术产业开发区转型，成为推进新型工业化发展的主力军。加快镇域经济发展，同步推进工业化、城镇化和农业现代化，重点规划培育发展50个城镇、1000个新型农村社区，因地制宜，突出特色，全面加快社会主义新农村建设。

　　（三）加快打造文化强市，建设更高水平的城市文明

　　推动文化大发展大繁荣，向现代海洋文化名城迈进，建设文化青岛。文化发展主要指标位居全国同类城市前列，建设区域性文化交流中心和文化产业中心。巩固文明城市创建成果，加强社会主义核心价值体系建设，大力弘扬微尘、红飘带精神，建设学习型、诚信型城市，塑造传承历史文化、突出地域特色、凸显时代要求、引领未来发展的城市精神，提升城市的精气神。大力发展公共文化服务，建设布局合理、服务便捷的基层文化设施，率先建成国家公共文化服务体系示范区。推动文化与科技、教育、体育、旅游融合发展，培育一批骨干文化企业，加快文化产业规模化、集约化、特色化发展。文化创意产业增加值占生产总值比重要达到10%，成为新的支柱产业。突出文化特色，培育文化精品，做强帆船之都、音乐之岛、影视之城等文化品牌。加强文化市场管理，建设文化环保城市，优化文化发展环境。

　　（四）加快发展民生事业，营造更加和谐的社会环境

　　努力保障和改善民生，促进经济社会协调发展，构建和谐青岛。更加注重公平正义和管理创新，更加注重百姓内心的幸福感受，让城乡居民学有优教、病有良医、老有颐养、住有宜居，成为宜居幸福城市的建设者和共享者。大力实施惠民工程，加快教育、医疗、文化、民政等千万平方米社会事业设施建设。全面实施中长期教育改革和发展规划纲要，实施教育设施建设双百工程，加快教育改革步伐，推进各类教育跨越发展，率先实现教育现代化。加快建设健康城市。实施医疗卫生设施百万工程和医学园区建设项目，新增病床1.2万张，居民主要健康指标达到全国领先水平，降低个人医疗卫生支出比例。大力发展体育事业，落实全民健身计划。完善公共就业服务体系，扩大就业规模，优化就业结构，构建和谐劳动关系。稳步推进收入分配制度改革，不断提高居民收入水平和消费能力。完善社会保险制度，大力发展社会救助、社会优抚、社会福利和慈善事业，健全覆盖城乡的社会保障体系。建立老年人福利普惠制度，完善社会养老服务体系。积极发展残疾人事业，提高残疾人康复和托养服务设施建设水平。完善多元化住房保障体系，扩大保障性住房建设规模，力争覆盖面达到20%。强化人口和计划生育利益导向政策体系，加快技术服务机构建设，提高人口出生质量。构建以社区为重点的基层社会管理服务体系，推进社会管理重心下移，提高社会管理科学化、法治化、民主化水平。深化平安青岛建设，加强公共安全体系建设，不断增强群众的安全感和对社会治安的满意度。做好双拥工作，支持国防建设和民兵预备役建设，巩固和发展同呼吸、共命运、心连心的军政军民关系。

　　（五）加快建设生态文明，打造更加宜居的美好家园

　　坚持绿色发展，争创国家生态园林城市，建设生态青岛。万元生产总值能耗和主要污染物排放总量达到国家和省的规定要求；市区污水集中处理率达到95%；完成市区河道治理；实现生活垃圾无害化处理；资源化利用率达到90%。对岸线资源和近海资源进行严格保护，把胶州湾建成全市人民的蓝色家园。加强生态建设，推进十绿工程，打造多层次城乡生态空间，森林覆盖率达到40%以上，创建国家森林城市。加强环保基础设施建设，强化资源节约和污染减排，高水平建设中德生态园、石化产业循环经济区等重点示范园区。发展节水型经济，建设节水型社会。办好2014年世界园艺博览会，让生活走进自然。

　　（六）加快推进改革开放，注入更加强劲的发展活力

　　加快重点领域和关键环节改革，建设活力青岛。推进国有企业和国有资产管理体制改革。深化财政、土地、户籍、价格、投融资、城市管理、城乡统筹、农村产权制度等领域的改革。降低民间资本准入门槛，放宽准入领域。加强中小企业公共服务平台建设，完善和落实对小微企业的扶持政策。健全要素市场体系，加大力度推进企业上市工作。完成国家级开发区体制机制创新试点和综合配套改革，提升各类园区发展水平。

　　加快转变对外经济发展方式。实施更加积极的开放战略，优化招商载体，创新引资方式，提高招商引资水平。加快外经贸优化升级，促进资本、人才、市场国际化和多元文化融合，形成更具竞争力的开放型经济。推进大通关工程，加快建设国际贸易中心城市，增进与国际友城的交流合作，提升国际经济合作层次，成为中日韩自由贸易区的重要前沿城市。按照国际惯例，营造更具竞争力的商务环境，进一步促进大开放，努力建设国际性城市。

　　各位代表！站在新的历史起点，新一届政府一定会增强使命感，抓住机遇、开拓进取，圆满实现五年奋斗目标，努力取得让青岛经济更发达、城市更宜居、文化更繁荣、社会更和谐、人民更幸福的新成就！

　　三、2012年主要工作安排

　　今年是全面落实市第十一次党代会精神的开局之年，也是实施“十二五”规划承前启后的重要一年。今年的工作，必须坚持突出主题、贯穿主线，稳中求进、好中求快，把稳增长、控物价、调结构、惠民生、抓改革、促和谐更好地结合起来，狠抓落实，奋力突破，努力开创经济社会发展新局面。主要预期目标是：全市生产总值增长11%，地方财政一般预算收入增长15%，固定资产投资增长18%，社会消费品零售总额增长16%，城乡居民收入增长11%，城镇登记失业率控制在4%以内，居民消费价格涨幅控制在4%左右，人口自然增长率控制在4‰以内。全面完成国家和省下达的节能减排各项目标任务。实际工作中，必须更好更快地完成各项任务。

　　（一）加快推进蓝色经济区建设，在蓝色引领、跨越发展上奋力突破

　　加快西海岸经济新区建设。深化西海岸经济新区规划，加快开发步伐。推进保税功能拓展区建设，提升出口加工区功能。整合岸线陆域资源，拓展董家口港区，加快配套设施建设，规划布局港口物流和临港大工业，支持青岛港转型发展、做大做强，建设区域性国际航运中心。做好青钢、海湾集团化工搬迁改造等项目前期工作。开工建设中德生态园10平方公里配套设施。

　　加快蓝色硅谷建设。推进10平方公里核心区和30平方公里配套区建设。加快海洋科学与技术国家实验室、国家深海基地、国家海洋设备检测中心、山东大学青岛校区等重点项目建设，促进高端要素集聚。推动中科院光电院青岛研发基地等项目建设，加快科技创新平台建设，打造蓝色经济科技支撑区、高端产业聚集区，争创国家创新型科技园区。

　　着力打造蓝色产业链。统筹规划布局蓝色经济园区、重点项目，推进传统临海、涉海产业改造升级，大力发展海洋生物医药、海洋新材料、海洋精细化工、海水综合利用、海洋高端装备制造等海洋新兴产业，拉长产业链，加快海洋产业规模化、集群化。加快董家口港区物流园、高新区蓝色生物医药产业园、崂山生物医药产业园、胶州蓝色高端制造业基地等海洋特色重点园区的建设。

　　（二）加快推进高端产业聚集区建设，在结构调整、产业升级上奋力突破

　　突出发展高端服务业。做好国家服务业综合改革试点工作。启动十项千万平方米的重点服务业工程。加快发展金融业，培育引进各类金融机构，促进金融与产业深度融合，建设崂山金家岭金融新区和即墨鳌山湾财富管理园区。组建青岛农商银行，重组海协信托公司。扩大基金规模，大力发展蓝色金融。切实防范和化解金融风险，营造良好的金融生态环境。加快发展现代物流业，出台进一步加快现代物流业发展的意见，推进一批重点物流项目建设，积极发展电子物流、冷链物流、供应链物流，建设高水平的公共物流信息服务平台。加快发展蓝色旅游业，发展青岛航空，开工建设邮轮母港，发展邮轮、游艇等新兴旅游产业，推进海上交通旅游集散中心建设，办好国际帆船周及系列国际帆船赛，申办世界杯帆船赛，打造海上旅游精品。办好中国国内旅游交易会、中美省州长旅游论坛等展会活动，推动旅游业向高端发展。发展现代商业，扩展海信广场商圈，加快华润中心、中铁青岛中心等大型高端商业综合体建设，发展高端时尚商业街区，打造青岛购物节消费品牌。办好国际啤酒节、国际消费电子博览会，培育会展经济知名品牌。加快发展科技服务业，建设高水平的科技信息公共服务平台，大力发展服务外包，加快建设千万平方米软件和服务外包产业园区，软件和信息服务业收入增长30%以上。加快发展中介服务业，积极引进培育融资担保、律师仲裁、认证检测、技术评估等高端中介机构，提高水平，打造品牌。鼓励和支持发展养老、健康、家政、物业等民生服务业。

　　大力发展高端制造业。制定全市工业园区布局规划，推进省级以上工业园区向高新技术产业开发区转型升级，促进新增工业功能区加快发展。促进优势产业集群发展，推动传统产业升级，大力引进产业龙头和配套项目，加快企业技术中心建设，实施1000项技术创新重点项目，加快技术改造和“两化”深度融合，打造一批在全国有影响力的特色产业链。

　　推动战略性新兴产业突破发展。制定战略性新兴产业发展指导目录，设立专项引导资金，促进物联网、云计算开发应用。推进高速列车制造基地、海洋仪器设备产业化基地等项目建设，发展新能源汽车产业，加快新能源材料产业园等园区建设。深入实施质量强市和品牌带动战略，推进国家高新技术产业标准化示范区建设。

　　强化项目和投入保障。把项目建设作为加快科学发展的生命线，集中力量建设总投资3800亿元的230个重点项目，确保全年完成投资800亿元以上，竣工投产70个项目。扎实推进300个工业转型升级项目，力争150个竣工投产。搬迁老城区工业企业10户，竣工6户。加强银企对接和项目推介，争取全年新增贷款超过1100亿元。

　　（三）加快推进现代农业发展，在农业增效、农民增收上奋力突破

　　抓好水利基础设施建设。以创建全国水利现代化试点城市为契机，加强农田水利建设，提高抗旱排涝能力。加快大沽河防洪主体工程建设。实施大水网工程，加速推进沐官岛水库建设。抓好农村规模化集中供水、高标准农田节水灌溉、病险水库加固、清洁型小流域治理等工程，确保防洪安全、水资源安全和水生态安全。

　　加大农业科技创新推广力度。深入开展 “农业科技促进年”活动。加强以种业为重点的农业科技创新，加强农业重大关键技术攻关和引进推广工作。健全基层区域性农技推广、动植物疫病防控、农产品质量监管等公共服务机构，完善农业科技服务体系。大力培训农村实用人才，培育一批有文化、懂技术、会经营的新型职业农民。

　　加快现代农业生产基地建设。加大“三农”投入，新增农业补贴重点向种养大户、农民专业合作社倾斜。推进“双百双千”工程，打造一批高水平的设施农业、规模农业、有机农业等特色农业基地和农产品出口加工基地。毫不放松地抓好粮食生产。加快国家级现代农业示范区建设。实施蔬菜、渔业等五大产业振兴规划。推进畜禽标准化规模养殖和传统养殖业退户进区。加强农业机械装备建设。强化农产品全程质量安全管理，大力发展高端品牌农业。支持龙头企业发展，提升农业产业化水平。

　　多渠道增加农民收入。落实强农惠农富农政策，增加农民转移性收入。加强农民职业技术培训，扩大农民转移就业规模，增加农民工资性收入。挖掘农业内部增收潜力，发展农村二三产业，扶持万名农民自主创业，增加农民经营性收入。健全土地流转服务体系，引导土地向家庭农场和种植大户集中，发展多种形式的适度规模经营，增加农民财产性收入。加大农村改革力度，完善农村土地政策，推进农村金融改革，创新农业组织形式，深化集体林权制度改革。实施新一轮扶贫开发，加大对经济欠发达村的帮扶力度。

　　（四）加快推进全域统筹，在三城联动、城乡一体上奋力突破

　　优化城市空间布局。完成新一轮城市总体规划修编、重点组团建设规划编制。加快西海岸经济新区和蓝色硅谷开发建设，推动中心城区转型提升。启动北部新城开发建设，打造科技、人文、生态新城。规划建设董家口临港经济区。加快大沽河流域生态经济区建设，打造惠及流域百万群众的民生工程。加快世园会筹办工作，推进世园生态都市新区规划建设。加快铁路青岛北客站建设，推进交通商务区建设。做好新机场定址建设前期工作，规划建设临空经济新区。

　　完善中心城区功能。完成全域轨道交通体系规划，推进城市地铁3号线建设，争取地铁2号线开工，做好连接西海岸经济新区、蓝色硅谷轨道交通线前期工作。加快青荣、海青和青连铁路、青龙高速等项目进度，完成胶州湾大桥接线等项目续建工程，推进新疆路快速路、福州路打通和重庆路改造等重点项目。规划建设一批大型停车场，加强停车秩序等专项治理，缓解停车难和交通拥堵问题。大力推进公共交通优先发展，加快建设城区智能交通管理服务系统。积极推进城乡公共交通一体化。加强统筹规划，加大对城市地下空间的开发利用力度。对老城区实施保护性开发，启动中山路区域旧城改造，打造历史文化旅游休闲区。重视和加快电网建设。新建、改造供水管网100公里、燃气管网230公里，开工10座天然气加气站，市区新增集中供热面积900万平方米。

　　推进城乡统筹发展。制定加快统筹城乡发展的意见，建立健全统筹城乡的财政、土地、投融资、社会管理等体制机制。坚持以村为基础、县为统筹，完成镇域产业发展规划、镇驻地开发建设规划和新型农村社区建设规划。设立镇级发展平台专项资金，实行镇级扩权改革，加快乡镇合并和新型农村社区建设，新型农村社区力争开工100个。优化农村公路网络，推动农村公路干道向镇和新型农村社区延伸。加快生态文明乡村建设，建设20个生态文明镇和60个示范村。提升县域经济发展水平。扩展五市工业功能区，提高承载能力，加快全市工业聚集发展。出台扶持政策，加快全域城镇和新型农村社区建设，加快四方、李沧旧城改造，推进平度、莱西市驻地向规模较大、实力较强的中等城市迈进。

　　（五）加快推进文化改革发展，在文化惠民、文化强市上奋力突破

　　加快发展文化事业和文化产业。深入实施公共文化惠民工程，推进市文化中心、市科技馆等公共文化设施规划建设，加快区市文化馆、图书馆、博物馆、档案馆和五市数字影院建设。全力做好十艺节各项筹备工作，办好第十届全国青少年小提琴比赛、全国指挥比赛。开工建设国家级青岛广告文化产业园，争创国家文化产业示范基地和国家数字出版基地。重视历史文化遗产保护，推进国家水下文化遗产保护青岛基地建设，做好全国可移动文物普查试点工作。加强对文化名人故居的保护，培育壮大文化人才队伍。加快国有文化企业改制步伐，打造国有文化企业“五朵金花”。实施创意青岛计划，申办联合国创意城市联盟音乐之都，举办世界城市音乐节、中国秧歌节。推进文化生产和传播方式创新，构建传输快捷、覆盖广泛的文化传播体系。深化文化市场综合执法改革，形成统一、规范、高效的文化市场管理格局。加强版权保护，打击各类侵权盗版行为。深入开展“扫黄打非”，净化文化环境。

　　提升城市文明程度和市民文明素质。做好第四轮全国文明城市创建工作，确保在全国测评中保持前列。深化“四心四德”工程，发挥社会课堂和乡村少年宫的育人作用，加强未成年人思想道德建设。繁荣发展哲学社会科学，加强科普工作，搞好法制宣传教育。依法管理民族宗教事务，促进民族团结。全面做好人口和计划生育工作，推行和谐幸福家庭促进计划，加快人口信息化建设。推动气象、地震、人防、统计、档案、史志、仲裁、红十字等事业协调发展。增强全民国防意识，加强民兵预备役建设，落实征兵优惠政策，为部队办好实事，增进军政军民团结。

　　（六）加快推进生态市建设，在节能减排、环境保护上奋力突破

　　推进节能减排。坚持调结构、控增量、减存量，建立完善能源消费总量和能源消费强度双调控机制，完成一批重点节能工程，确保全市万元生产总值能耗下降3.7%。抓好建设、交通运输等领域污染减排，加强工业点源、农业面源和生活污染源治理，推进农村污水、垃圾集中收集处理，提高城镇污水处理设施水平。制定实施低碳城市发展规划，大力发展绿色产业。培育高效节能产品、先进环保设备和节能环保产业，加强电动汽车应用推广工作。推进中水回用，做好海水淡化一体化循环经济试点，完善再生资源回收利用体系，建设“城市矿产”示范基地。办好第二届中国国际循环经济成果交易博览会。积极推进低碳交通运输体系试点城市建设，新增800辆节能环保型公交车。

　　加强环境保护。深入开展大干300天市容环境整治行动。推进市区河道污染整治，基本完成海泊河整治，做好李村河、张村河前期整治工作。加强胶州湾、饮用水源地、风景区的生态保护，建成具有国际先进水平的城市供水水质监测中心。突出抓好大气污染防治，实施市区汽车尾气治理。制定细颗粒物（PM2.5）监测方案，开展治理工作。启动创建国家森林城市工作，实施植树增绿工程，建设连片万亩林场，抓好交通干道和市区路网绿化，搞好海防林和水系绿化。推进浮山综合整治和太平山中央公园建设，新建改建绿地660万平方米，大幅度增加生态绿量。实施最严格的耕地保护政策，严格执行投资项目用地、节能、环保、安全等准入标准，杜绝高耗能、高污染、低水平项目。加快地下和山体采矿业退出，保护生态地貌，努力修复和保护山青水绿、碧海蓝天的宜居生态环境。

　　（七）加快推进改革开放，在体制创新、开放转型上奋力突破

　　推进改革取得实质性进展。制定实施经济体制改革指导意见。深化国有企业改革，有序推进国有资本逐步退出一般竞争性领域，强化行政事业国有资产监管。充分发挥工商联和各类商会作用，落实促进非公有制经济发展的各项政策措施，大力发展民营经济，鼓励进入社会事业等领域发展。加大对小微企业特别是创新型小企业的支持力度，帮助小微企业减负增效。推进企业信用体系建设。建立企业破产预警系统。深化投融资体制改革，推动政府投资公司向匹配战略、主业突出、管理现代、带动力强的现代企业转型。系统整合市政类企事业，建设运营高效、支撑力强的企业实体。要下大决心、用大气力支持企业上市融资，大规模突破资本市场。加强政府性债务管理。深化财政体制改革，健全财权与事权匹配的财政运行机制，优化财政支出结构，加快向公共财政转型步伐。改进财政资金投入方式，完善市场机制，更多地利用社会资源推进城市建设。加强政府投资项目预决算管理，提高财政资金集约利用水平。实施财源建设倍增计划，发展财源型经济，增强财政支撑能力。加快推进政府改革。转变政府职能，坚定不移地加快推进政企分开、政事分开、管办分离，更好地发挥市场主体的作用和市场配置资源的基础性作用。创新运行模式，建立高效运作的重点区域发展体制。深化行政审批制度改革，减少审批项目，提高审批效率，全市项目立项、环评、规划、土地、开工等投资类行政审批服务量要比去年提高30%。改进政府服务方式，推动公共服务社会化，完善以服务对象为导向的公共服务评价机制。完善目标考核体系，健全工作推进机制。积极稳妥地推进事业单位改革。深化价格改革，完善资源性产品价格形成机制。

　　提高对外开放的质量和水平。实施外贸转型升级行动计划，保持对外贸易稳定发展。加快特色出口基地建设，扩大自主研发、自主品牌、自主营销产品出口，保持传统市场份额，深度开发新兴市场，进出口总额增长10%。提升发展货物贸易，加快发展服务贸易，突破发展转口贸易，推动传统对外贸易向现代国际贸易转变。推进服务外包倍增计划。实施外资赶超行动计划，提高利用外资质量。紧盯世界500强和跨国大公司，以蓝色经济和战略性新兴产业为重点，引进100个先进制造业大项目、100个现代服务业大项目。完善招商工作机制，健全境外招商工作网络，组织高层次招商推介活动，在日、韩、德和我国香港等地设立商务代表处，加强海峡两岸经贸合作，推进中日韩经济合作实验区建设。实施开放园区提升计划，增强资源聚集能力。支持保税港区带动出口加工区转型发展，加快中德生态园规划建设进度，继续规划建设多元化的国际经济合作区。支持企业“走出去”，到境外建设生产基地，开发资源和市场，设立研发中心，收购品牌和营销网络，参与全球经济合作，提高国际竞争力。开通青岛至欧美直达航线。创新对口支援合作模式，促进对口支援地区经济发展、民生改善。扩大对外宣传，提升青岛品牌的国际影响力。

　　（八）加快推进创新型城市建设，在创新驱动、人才强市上奋力突破

　　着力提升自主创新能力。深入实施国家技术创新工程试点，完善城市创新体系。突出科技创新平台载体建设，启动千万平方米孵化器工程建设，加快各区市省级开发区孵化器建设。推进中科院在青院所及研发基地建设，加快引进中船重工710所、中国家用电器研究院等研发机构。加快发展实验室经济，推进5家企业国家重点实验室、7家国家工程技术研究中心和海洋工程防腐等公共研发平台建设，组建5家产业技术创新战略联盟，加快企业技术中心建设。推广四方橡胶行业产学研示范基地模式，创新产学研资合作机制，制定更优惠的政策，鼓励高校、科研院所科技人员进入孵化器和企业创新创业。重点培育具有核心竞争力、发展潜力大的创新型企业，力争创新型企业超过250家。组织实施科技发展重大专项，开展70项关键技术攻关。促进科技与金融融合发展，建立科技信贷风险准备金，加强对科技型中小企业的投融资服务。出台促进专利工作发展的意见，大力实施知识产权优势企业和优秀人才培育工程，建立专利产业化基地，搭建知识产权公共服务平台，力争发明专利申请量达到7000件，增长30%以上。深入实施标准化战略，鼓励企业创新成果成为行业标准、国家标准、国际标准。

　　全面加强人才工作。加大人才引进力度，大力实施“443引才工程”和蓝色人才集聚计划、青岛英才计划等人才工程，筹建中国海洋人才市场，办好蓝色经济国际高端人才项目洽谈会，着力引进蓝色经济人才、紧缺急需人才、高层次领军人才和创新型团队。抓好专业技术人才、企业经营管理人才等各类人才队伍建设，做好人才管理服务工作。创新人才政策机制，推进人才特区建设，启动千万平方米人才公寓建设，建立政产学研一体化人才公共服务平台，制定出台更加灵活、开放、有效的人才政策，鼓励创新，宽容失误，营造一流的创新创业环境，全力加快建设创新型城市。

　　（九）加快推进社会建设，在服务群众、改善民生上奋力突破

　　着力解决群众最盼、最急、最忧的问题，努力提高群众的幸福感。今年计划安排民生投入163亿元，实施教育、医疗、文化、民政等社会事业设施千万平方米工程，在办好10件市办实事的同时，进一步提高民生保障水平。

　　大力支持教育优先发展。全市财政性教育支出占财政支出比重提高到17%，加强城乡统筹，推进教育均衡。加大学前教育投入力度，建立公办、普惠性民办幼儿园生均财政拨款制度，对五市新建、改扩建公办幼儿园给予补助，规范学前教育收费。免除全市义务教育阶段学生教科书和作业本费。统筹各级各类教育发展，优化中小学、幼儿园布局，开展农村200所幼儿园标准化建设，完成农村薄弱中小学改造，增加居民区中小学和幼儿园配套。加大专用校车配置力度，建立专用校车政府补贴制度。以现代化、规范化为办校标准，统筹启动七区高中和职业学校建设。创新机制，加快发展五市高等职业技术教育。推进国家高职综合改革和省统筹职业教育发展改革等试点，探索创新型人才培养模式。

　　着力推进医药卫生事业改革发展。以破除以药养医为抓手，深化公立医院改革，扩大基本药物制度实施范围，取消以药补医，严格实施药品、器材集中采购，精心诊疗，科学用药，控制费用总额，严厉惩治处方和采购中的商业贿赂，降低药品费用比重。加快人事分配制度改革，能上庸下，多劳多得，调动医护人员积极性。规范开展医师多点执业试点。推进县级医院综合改革。鼓励民办公助多元化办医。完善新农合制度，最低筹资标准提高到每人每年300元。完善财政、价格和医保补偿机制，全面推行基层门诊费用报销。免费实施儿童口腔疾病基本预防。实施先看病后付费制度。提高紧急救助能力。深化基层医疗卫生机构综合改革，促进城乡统筹，加强基层全科医生、乡村医生等人才队伍建设。推进县级医院和市第三人民医院、市妇女儿童医院二期、市立医院东院区二期、市公共卫生医疗中心、市精神卫生中心等项目建设。

　　努力增加就业改善生活。实施更加积极的就业政策，统筹做好高校毕业生、农村富余劳动力和困难群体就业，新增城乡就业25万人。完善创业扶持政策体系，实施大学生创业引领计划，完善创业孵化基地运行机制，创建国家级创业型城市。扩大公益服务采购规模。推进市级家庭服务业培训基地建设，提升实训能力。加强企业工资分配宏观调控，积极推行工资集体协商制度，引导企业建立职工工资正常增长机制，促进城乡居民收入实际增长和经济增长保持同步。扶持农产品生产流通，保持物价基本稳定。加快发展连锁经营，推进市区农贸市场建设。广泛开展全民健身运动，新建一批健身园和健身路径，改善居民健身条件。

　　加快完善社会保障体系。推进社会保险扩面征缴和市级统筹。提高城乡低保和基本养老金标准，完善社会救助和保障标准与物价上涨联动机制。实施市社会福利院和儿童福利院改扩建项目，开工建设市盲校新校和市中心聋校扩建工程，推进市优抚医院等项目建设。建立大病医疗救助制度。新建9处敬老院，提升敬老院设施和服务水平。探索建立老年护理保险、养老机构意外伤害保险和高龄津贴制度，大力发展医养结合的机构养老、社区养老和居家养老，重点解决困难老人、空巢老人和失能老人的生活照料和医疗康复问题。对妇女住院分娩实施补助，免除死亡居民基本殡葬费。推行残疾儿童学前教育免费制度，实施残疾人康复救助服务和困难重度残疾人托养服务工程。

　　继续搞好保障性住房建设和房地产市场调控。制定加快公共租赁住房建设意见，健全多元化住房保障体系，努力让更多的创业者安居乐业，让更多的中低收入者住有所居。鼓励企业参与公共租赁住房建设。开工建设保障性住房2.3万套，配租配售保障性住房9000套。推进城中村和旧城区改造，新开工项目25个，回迁项目18个、惠及居民8000户。新建农村住房2.5万套，改造危房4060套。完善保障性住房建设、分配、管理、退出制度。巩固房地产市场调控成果，抑制投机、投资性需求，加大普通商品房供应，推动房价合理回归，促进房地产市场长期平稳健康发展。大力推广绿色建筑，注重提高住房品质。

　　加强和创新社会管理。推进基层社会管理服务中心建设，推行网格化服务管理模式。培育壮大各类社会组织，发挥好人民团体、基层自治组织、社会组织和企事业单位的协同管理作用。稳妥推进户籍管理制度改革，探索实行居住证制度。加强和改进对流动人口的管理和服务，促进流动人口有序融入城镇。做好重大事项和重点项目社会稳定风险评估，从源头上防范和化解社会矛盾。深入开展矛盾排查调处工作，完善大调解工作格局。畅通信访渠道，健全群众利益诉求表达机制，着力化解信访积案。重视做好刑释解教人员的社区矫正、职业培训、就业辅导、融入社会工作。深化平安青岛建设，加强社会管理综合治理，健全完善打防控一体化的治安动态防控体系，加强社会治安重点地区排查整治工作，依法惩处各类犯罪，增强群众安全感。加强网络舆情引导，营造健康的网络环境。加强应急体系建设，提高城市应急能力，增强公众防灾减灾自救互救能力。强化安全生产监督管理，实行安全月安全周制度，严格执法、严格监督、严格考核、严格纠责，淘汰事故易发落后产能，遏制重特大事故发生。完善食品安全管理体系，建设肉类蔬菜流通追溯体系，严格市场主体准入、严格生产营销监管，提高食品安全水平。

　　四、改进和加强政府自身建设

　　各位代表！全心全意为人民服务是人民政府的根本宗旨，扎扎实实为群众谋利益是各级政府及全体政府工作人员的应尽职责。必须牢固树立发展为了人民、发展依靠人民、发展成果由人民共享的理念，把惠民富民作为政府施政的根本目标，尽心尽力为人民群众谋福祉，努力让青岛人民的生活更加美好幸福！ 四、改进和加强政府自身建设 各位代表！建设宜居幸福的现代化国际城市，政府工作必须有更大作为；顺应人民群众过上幸福生活的新期待，政府工作人员必须付出更大努力。要增强率先意识、忧患意识和创新意识，寻标、对标、达标、夺标，自我加压，尽心履职，努力建设人民满意的服务型政府。

　　（一）自觉坚持依法行政

　　围绕建设法治政府，加强和改进政府立法，提高依法行政水平。认真执行市人大及其常委会的决议、决定，坚持重大事项向人大报告、向政协通报制度，主动接受人大法律监督、工作监督和政协民主监督。切实做好人大代表建议、意见及政协提案办理工作，定期通报办理情况。认真听取各民主党派、工商联和无党派人士意见。充分发挥行风在线、民生在线、政务服务热线、网络在线问政、纠风网等互动平台作用，继续开展向市民报告、听市民意见、请市民评议活动，虚心接受社会监督，及时回应群众诉求。健全科学民主决策机制，把公众参与、专家论证、风险评估、合法性审查、集体讨论决定作为行政决策的必经程序。贯彻实施《山东省行政程序规定》，深入开展“行政程序年”活动。针对行政执法中存在的突出问题，加强法规和制度建设，依靠健全的机制规范执法行为。规范行政处罚自由裁量权，推进行政处罚权规范透明运行，促进公正文明执法。严格落实《行政强制法》，依法规范实施主体，严格遵守行政强制程序，强化对实施行政强制的监督。创新行政调解体制，完善行政复议机制。大力发展电子政务，深化政府信息公开，推进行政权力和公共服务网上公开透明运行。加强政务诚信建设，切实提高政府公信力。各级行政机关和公职人员都要切实增强法治意识，带头自觉守法，严格依法行政，决不允许任何单位、任何人凌驾于法律之上。

　　（二）努力做到勤政廉政

　　强化各级政府作风建设，牢固树立正确的价值观，正确对待权力、政绩和地位，力倡干事、反对无为，力倡创新、反对僵化，力倡团结、反对内耗，力倡高效、反对扯皮，力倡务实、反对虚漂，力倡清廉、反对腐败，心底无私天地宽，奉公为民可担当，努力营造能干事、可成事的清明发展环境和政治生态。始终坚持从群众的利益出发，真诚倾听群众呼声，真实反映群众愿望，真情关心群众疾苦。严格执行领导干部廉洁从政准则等各项规定，加强廉政文化建设和警示教育，强化岗位廉政风险防控。健全完善惩治和预防腐败体系，各级政府领导成员要带头履行反腐倡廉责任，努力保持自身的先进性和纯洁性。加强行政审批、行政处罚、公共资源交易等领域的电子监察，推进政府投资项目、财政专项资金、企业国有资产等领域的网络化监管。强化工程建设领域突出问题专项治理，加强政府投资项目决策与实施的监督检查。推进公共资源交易监、管、办分离改革，统一纳入平台交易和监督。坚持节俭办事，压缩“三公”支出，细化预算编制，严格审计监督。落实《青岛市预防职务犯罪条例》，建立关键岗位公开监督廉政制度，坚决查处失职渎职、贪污贿赂、以权谋私和充当黑恶势力保护伞等违法违纪案件，坚决纠正损害群众利益的不正之风。廉洁出勇气，廉洁出效率，廉洁出业绩，要始终严于自律，努力建设亲民为民、务实高效、清正廉洁的人民政府。

　　（三）全面加速提升创新增效落实

　　深入贯彻市第十一次党代会精神，解放思想，创新突破，赶超跨越。要治理庸、懒、散、慢、拖、瞒问题。加速，就是要抢抓机遇、只争朝夕，提高政府工作效率；提升，就是要以世界眼光、国际标准衡量工作，提高素质、增强能力，提升发展水平；创新，就是要以创新的思维和改革的办法破难题、攻难关，创先争优求突破；增效，就是要集中心智和力量，干成事、出效果，提高行政效能；落实，就是要科学周密地决策、雷厉风行地执行、百折不挠地推进，践行承诺、敢于担当、奋发有为，全力开创各项工作的新局面。

　　各位代表！新一届政府的履职，依靠的是党的坚强领导和人民群众的信任支持。让我们更加紧密地团结在以胡锦涛同志为总书记的党中央周围，在中共青岛市委的坚强领导下，与全市人民一道团结奋斗、开拓进取，率先科学发展、实现蓝色跨越，加快建设宜居幸福的现代化国际城市，以优异成绩迎接党的十八大胜利召开！
